# Complete Genome Sequence of a Mosaic NADC30-Like Porcine Reproductive and Respiratory Syndrome Virus in China

**DOI:** 10.1128/genomeA.01428-16

**Published:** 2016-12-22

**Authors:** Lin-jian Wang, Zhenhua Guo, Songlin Qiao, Xin-xin Chen, Gaiping Zhang

**Affiliations:** a College of Life Sciences, Henan Agricultural University, Zhengzhou, People’s Republic of China; b Key Laboratory of Animal Immunology of the Ministry of Agriculture, Henan Provincial Key Laboratory of Animal Immunology, Henan Academy of Agricultural Sciences, Zhengzhou, People’s Republic of China; c Jiangsu Co-Innovation Center for Prevention and Control of Important Animal Infectious Diseases and Zoonoses, Yangzhou, People’s Republic of China

## Abstract

Here, we report the complete genome of an NADC30-like porcine reproductive and respiratory syndrome virus (PRRSV) strain, HNhx, which was isolated from Henan Province, China, in 2016 and was characterized by recombination with JXA1 strain (an epidemic highly pathogenic PRRSV strain in China) in Nsp4 to Nsp9.

## GENOME ANNOUNCEMENT

Porcine reproductive and respiratory syndrome (PRRS) has caused enormous economic losses since its emergence in the United States in the late 1980s (1). The causative agent, PRRS virus (PRRSV), is a single positive-strand RNA virus and is approximately 15 kb in length (2). According to the genetic and antigenic characteristics, PRRSVs can be divided into two major genotypes: European type (type 1) and North American type (type 2). Recently, several provinces in northeastern and central China had reported the outbreaks of NADC30-like PRRSVs, which shared high nucleotide similarity with NADC30 isolated in the United States in 2008 (3–5). Here, we report the complete genome sequence of the NADC30-like variant PRRSV HNhx.

The HNhx strain was isolated from the tissue sample of diseased pigs collected from one pig farm with clinical outbreak of porcine reproductive and respiratory syndrome (PRRS) in 2016. Full-length HNhx genome amplification was performed with six special primer pairs by reverse transcription-PCR (RT-PCR). The PCR products were purified and subcloned into T-vector pMD19 (simple) (TaKaRa). The 5′ untranslated region (UTR) and 3′UTR regions were obtained by rapid amplification of cDNA ends (RACE) using the SMARTer RACE 5′/3′ kit (Clontech). For each product, three independent clones were sequenced and then assembled into the full-length genome of HNhx using the DNAStar (Lasergene) software. The result showed that HNhx was 15,019 nucleotides (nt) in length, excluding the poly(A) tail. Similar to other NADC30-like PRRSVs, HNhx contained a discontinuous 131-amino-acid (aa) deletion in Nsp2 identified as a 111-aa deletion at positions 323 to 433, a 1-aa deletion at position 483, and an 19-aa deletion at positions 501 to 519. Genetic analyses showed that the complete genome of HNhx exhibited highest identity (92.91%) with NADC30 strain compared to all the other available PRRSV strains deposited in the NCBI and shared 81.66%, 83.31%, 82.77%, and 83.37% identity with VR2332, CH-1a, HB-1(sh)/2002, and JXA1, respectively.

Recombination events were determined with SimPlot version 3.5.1 and the Recombination Detection Program 4 (RDP4). The data revealed that HNhx was the result of recombination between the NADC30 virus and highly pathogenic PRRSV JXA1 strain. The recombined region located in Nsp4 (nt 5296) to Nsp9 (nt 7994). These genomic characteristics of the HNhx strain would provide valuable genomic data for elucidating the evolutionary relationship of PRRSVs in China.

### Accession number(s).

The complete genome sequence of the HNhx strain is available in GenBank under the accession number KX766379.
